# Influence of Female Sex Hormones on Ultra-Running Performance and Post-Race Recovery: Role of Testosterone

**DOI:** 10.3390/ijerph181910403

**Published:** 2021-10-02

**Authors:** Eladio Collado-Boira, Pablo Baliño, Ana Boldo-Roda, Ignacio Martínez-Navarro, Bárbara Hernando, Paula Recacha-Ponce, Carlos Hernando, María Muriach

**Affiliations:** 1 Faculty of Health Sciences, Jaume I University, 12071 Castellon, Spain; hernandb@uji.es (B.H.); recacha@uji.es (P.R.-P.); muriach@uji.es (M.M.); 2 Obstetrics and Gynecology, University Hospital of La Plana, 12540 Vila-Real, Spain; anaboldoroda@gmail.com; 3 Department of Physical Education and Sport, University of Valencia, 46010 Valencia, Spain; ignacio.martinez-navarro@uv.es; 4 Sports Health Unit, Vithas-Nisa 9 de Octubre Hospital, 46015 Valencia, Spain; 5 Sport Service, Jaume I University, 12071 Castellon, Spain; hernando@uji.es; 6 Department of Education and Specific Didactics, Sport Service, Jaume I University, 12071 Castellon, Spain

**Keywords:** ultra-endurance, hormones, female athletes, physical performance, muscle damage, muscle fatigue, hormonal contraception

## Abstract

In recent years, increasing numbers of women have participated in extremely long races. In adult males, there is a clear association between physiological levels of endogenous sex hormones and physical performance. However, the influence of plasmatic sex hormones and the effects of different types of hormonal contraception (HC) on the modulation of physical performance in adult females remain to be fully clarified. Eighteen female ultra-endurance athletes were recruited to participate in the study. Different variables were studied, including hematological parameters, body mass index, and body composition. Strength measurements were obtained using the squat-jump and hand-grip test. A repeated-measures analysis demonstrated significant differences in hematological values of CK and LDH pre-race as compared to immediately post-race and after 24/48 h. Furthermore, statistical differences were found in squat-jump and hand-grip test results after the ultramarathon. Testosterone, estradiol, and the testosterone/estrogen ratio were significantly correlated with muscle fatigue and were found to be indirect markers of muscle damage. A multivariate analysis demonstrated the protective role of testosterone against muscle damage and severe fatigue. Fluctuations in endogenous testosterone levels were correlated with greater fatigability and muscle damage after the competition. Adjusting the menstrual cycle with HC would not provide any further benefit to the athlete’s competitive capacity.

## 1. Introduction

Ultramarathon races are defined as sporting events that involve running and/or walking for distances greater than the official marathon distance of 42.195 km. In recent years, these competitive events have increased in popularity [1]. These extremely long races defy our physiological systems, inducing muscle injuries, respiratory fatigue, and cardiac and renal damage. They, thus, provide an outstanding model to evaluate the effects of ultra-endurance activities on the human body. The percentage of women participating in ultra-endurance sports has greatly increased in recent years [2]. In elite athletic competitions, males and females are usually separated, thus avoiding disadvantages for females in terms of strength, power, and speed as compared to their male counterparts.

It has been demonstrated that sex differences in sports performance are associated with higher circulating testosterone concentrations in males during puberty. In fact, results from sports competitions show no sex differences prior to puberty. Postpubertal testosterone-induced changes occur with regard to muscle mass, strength, bone composition, and hemoglobin levels, conferring a physical advantage in sporting events [3].

Male sex hormones such as testosterone, dihydrotestosterone, and dehydroepiandrosterone modulate the different physiological mechanisms that are responsible for athletic performance improvement [4,5]. Testosterone binding to the androgen receptor at the surface of the muscle fiber increases calcium release from the intracellular stores, activating different mechanisms that increase the number of muscle fibers and satellite cells, as well as the size of motor neurons [6]. Thus, skeletal muscular activity is enhanced, eliciting improved athlete performance and recovery [7].

Although there is a clear association between physiological levels of endogenous testosterone and physical performance in men [3,8], the influence of plasma concentrations of female androgens on sports performance needs to be clarified. The paucity of research on female athletes can be attributed to the cultural marginalization of women in sport and a lack of female participation in both the research and sporting contexts [9]. It is also the result of the cofounding variables of female sex hormone fluctuations, for example in terms of the endogenous hormone profile and differences in this profile under the use of hormonal contraception [10]. Further, the greater incidence of anovulation and luteal phase deficiency in female athletes complicates research in this regard [11].

Athletic amenorrhea is common in elite female athletes. It has been postulated to be an adaptative mechanism to a negative energy balance (hypometabolic state), and is associated with low testosterone levels [12]. Conversely, Hagmar [13] reported that most cases of menstrual disturbances observed among female elite athletes were due to polycystic ovary syndrome (PCOS). Interestingly, this syndrome is characterized by an elevated ovarian production of testosterone and is associated with greater muscle mass [14,15]. This increase in testosterone levels may positively modulate physical performance in athletes, especially in ultra-endurance events. 

Estrogen is thought to be another hormonal factor modulating muscle strength, metabolism, and stiffness [16]. Moreover, the testosterone/estrogen ratio (T/E ratio) has been recently considered as a predictor of over-training syndrome in male athletes [17]. Hence, basal estrogen levels and the testosterone/estrogen ratio (T/E) are critical to a better understanding of ultra-trail performance and post-race recovery in female runners. 

It has been reported that many elite female athletes use hormonal contraception, with figures varying from 20% to 70% depending on the country and sport [18,19,20,21]. However, little is known about the impact and prevalence of HC use and the effects of intermittent treatment. Furthermore, the physiological aspects of HC use are not always considered when monitoring the health of athletes. 

As such, we hypothesized that the menstrual cycle phase and, in particular, its influence on testosterone levels could modulate muscular physiological response and recovery after intense sporting activity in ultra-trail events. Thus, we aimed to elucidate the influence of basal testosterone and the T/E ratio on the post-race loss of skeletal muscle strength and muscle damage in female ultra-trail runners. The objectives of this study were extended to consider whether HC could influence these variables.

## 2. Materials and Methods

### 2.1. Study Design

This was a cross-sectional observational study that formed part of the Penyagolosa Trail Healthy Women project and involved a sample of 18 amateur runners who participated in the Penyagolosa Trail CSP ultra-trail race on 12 May 2018. The trail track was of 107.4 km, with an incline of 5604 m and a decline of 4356 m. All subjects were fully informed of the procedure and gave their written consent to participate. For more detailed information on the methods, see the study registration information in the ClinicalTrails.gov database (registration number NCT03990259).

### 2.2. Study Population and Ethical Approval

The sample was composed entirely of amateur runners. In total, 35 women finished the race. Our sample size included 18 female athletes, representing 51.42% of the total sample finishers. This sample can be considered representative, with a 95% confidence interval, a precision of 6%, and a proportion of 3%. 

The mean age was 41+/–6 years, with an average height of 1.61 ± 0.05 centimeters and weight of 56.92 ± 4.36 kilograms. In relation to training habits, the runners underwent 4.87 ± 0.9 days of weekly training, with an average training time of 9.07 ± 2.54 h and distance of 73.07 ± 43.32 kilometers. The average length of menstrual bleeding was 3.56 ± 1.04 days, and a total of 6 participants in the study used some type of hormonal contraception (2 used oral contraceptives, 1 used a vaginal ring, and 3 used hormonal IUDs).

The investigation was conducted according to the Declaration of Helsinki, and the project was approved by the Research Ethics Committee of the university (file number CD/007/2019). Informed consent was obtained from all subjects participating in the study.

### 2.3. Hematological Variables

Blood samples were collected from an antecubital vein by venipuncture on the same day of the race prior to starting, after crossing the finishing line, and 24 and 48 h later. The serum hormones related to the menstrual cycle used in the present investigation were estradiol and testosterone. Lactate dehydrogenase (LDH) and creatine kinase (CK) were used as indicators of muscle membrane disruption resulting from tissue injury. The hematological variables included ferritin, hemoglobin, hematocrit, and red blood cell count. The biochemical results obtained immediately post-race were adjusted by employing the Dill and Costill method [22], using hematocrit and hemoglobin to determine the magnitude of plasma volume changes after the race in each participant.

### 2.4. Body Mass Index (BMI) and Body Composition Assessment

Prior to starting on the same day as the race we measured the height and weight of all volunteers. They were also subjected to a body composition evaluation test (Tanita BC-780MA, Tanita Corp., Tokyo, Japan).

### 2.5. Loss of Strength Measurement

To assess the force, speed, and power of the extensor muscles in the lower extremities we used the squat-jump (SJ) test. The SJ test has been validated and is based on 3 simple parameters (body mass, jump height, and push distance) [23].

Grip strength (HG) is a direct measure of hand skeletal muscle strength. It is an index of endurance and general muscle capacity, and can reflect the association between peripheral strength and exercise capacity [24].

Previous studies have also suggested that the strength decline index (SDI), calculated as the decline in strength as a proportion of baseline values (measured through tests such as the HG and SJ), is a useful assessment of muscle fatigue [25].

Briefly, volunteers were first familiarized 3 to 5 times with the technical aspects of the testing procedures. The HG and SJ tests were performed before the race and 15 min after the race. For the HG assessment, volunteers remained in standing position, holding the grip dynamometer (T.K.K. 5401 GRIP-D, Takei Scientific Instruments Co., Tokyo, Japan) in their dominant hand. They were asked to squeeze the dynamometer for 5 s and the test was performed twice, with 30 s of rest in between attempts. The peak value for each individual was retained for statistical analysis.

For the SJ, the participants were asked to jump as high as possible. In the starting position, hips and knees were flexed 80° and hands were immobilized on hips to avoid arm swing. Jump height was estimated by the flight time as measured with a contact platform (Chronojump, Barcelona, Spain). The test was performed twice, with 90 s of rest between attempts. Each individual’s best performance value was retained for statistical analysis [26].

### 2.6. Statistical Methods

The level of significance was established at *p* < 0.05. The data are presented as the mean ± standard error of the mean (SEM). We opted for non-parametric analysis [27] due to the sample size (*n* < 30). Spearman’s correlation analysis was used to assess whether the baseline values of sex hormones were interrelated or related to the loss of upper (HG) and lower limb (SJ) strength, hematological variables representing muscle membrane disruption (CK, LDH), and the percentage of muscle mass (MM). For each subject, values for the variables HG, SJ, MM, CK, and LDH post-race and 24 and 48 h later were related to the individual baseline levels to define the delta scores (Δ): Δ (fold increase) = (post-race value—baseline value)/baseline value [28].

Test outcome meaningfulness was estimated through the size of the estimated effect of the correlation: strong, moderate, and small (>0.5, 0.3–0.5, and <0.3, respectively). 

Regarding the use of HC method, the strength and muscular damage variables (expressed as a delta score of the baseline values for each subject) were compared using the Mann–Whitney U test. The test outcome meaningfulness was estimated through Cohen’s d effect size pairwise comparisons. A Cohen’s d value < 0.5 was considered small, while a value between 0.5 and 0.8 was considered moderate, and a value greater than 0.8 was considered as large.

Finally, multiple regression analysis was performed using the forward stepwise method. Only normally distributed variables were used as dependent variables. Among the models obtained, the parsimony principle was applied [29]. Given the limited simple size and the non-normal distribution of independent variables, residual errors from the resulting models were inspected to ensure their normal distribution and, thus, the reliability of our regression models [30]. To identify the predictive value of the model, the Cohen criterion [31] was applied to one-way ANOVA models. This criterion indicates that R^2^ values less than 0.10 do not present a relevant explanatory value, while R^2^ values between 0.10 and 0.25 indicate a dependence of the explanation of the variance of the analyzed variable on the identified factors, and with R^2^ values above 0.25, we can affirm that the explanatory model is very clinically relevant.

## 3. Results

The average race finish time was 22 h 20 min ± 2 h 24 min. Data regarding sex hormones and variables of muscle fatigue, systemic inflammation, and damage to muscle tissues are shown in Table 1. Repeated-measures analysis demonstrated significant differences in CK and LDH values pre-race as compared to immediately post-race and 24/48 h later. Similarly, significant differences were found between the loss of muscle strength measured with the HG, SJ, and loss of muscle mass after the ultramarathon. Baseline values of sex hormones, fatigue, and muscle strength were within the normal clinical range. 

**Figure 1:**
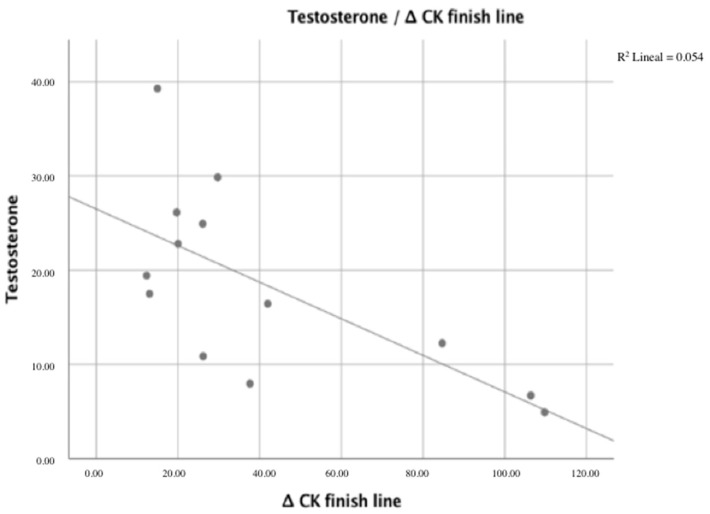
Correlation between ∆ CK finish line and testosterone levels.

**Figure 2:**
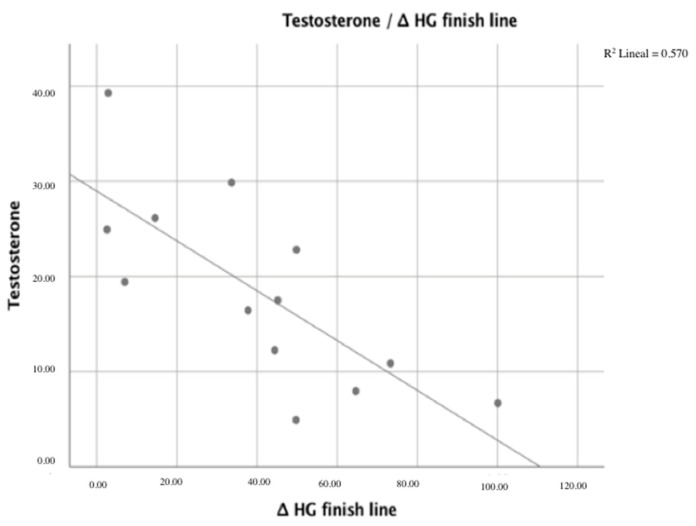
Correlation between ∆ HG finish line and testosterone levels.

Spearman’s correlations between sex hormones, muscle fatigue variables, and indirect markers of muscle damage are listed in Table 2. We analyzed the correlation coefficients to determine whether testosterone and estradiol were correlated with muscle fatigue variables and indirect markers of muscle damage. The correlation coefficients of muscle membrane disruption for Δ CK finish line, Δ CK 24 h, Δ CK 48 h, Δ LDH 24 h, and Δ LDH 48 h were −0.674, −0.585, −0.619, −0.585, and −0.615, respectively, indicating a correlation with testosterone levels (*p* < 0.05). Moreover, a correlation was found between Δ HG finish line and testosterone levels (*p* < 0.05), as shown in Figure 1 and Figure 2. The correlation coefficient was −0.755. 

**Figure 3:**
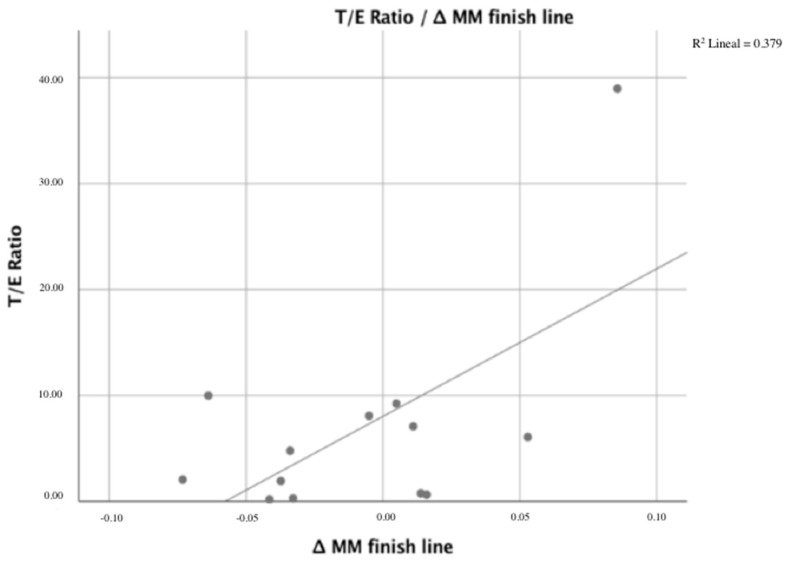
Correlation between ∆ MM finish line and T/E ratio.

Results of the testosterone/estrogen ratio demonstrated a correlation with LDH and loss of MM (Figure 3) for the following finish-line parameters: Δ LDH finish line, Δ LDH 24 h, Δ LDH 48 h, and Δ MM finish line (*p* < 0.05). The correlation coefficients values were 0.581, 0.595, 0.620, and 0.625, respectively. 

**Figure 4:**
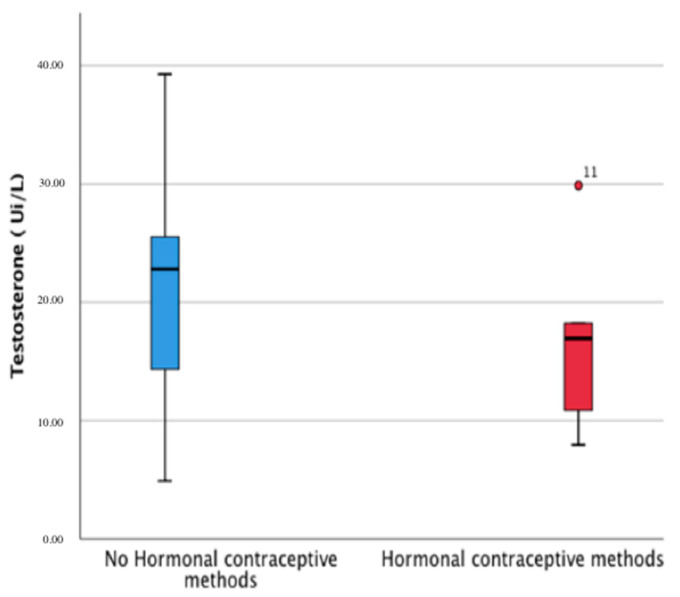
Testosterone levels (IU/L) in the non-HC and HC method groups.

The relationship between HC groups and the study variables is shown in Table 3. An HC method was used by 30.76% of female runners. No significant differences were found between the groups in the study (Figure 4). Regarding the use of the HC method, estradiol levels differed statistically between HC and non-HC users (33.10–32.83 and 129.20–93.85; *p* < 0.05, respectively). Interestingly, although the Mann–Whitney U test did not show significant differences, a tendency was found between groups for all the serum concentration hematological parameters.

Results from a multivariate analysis are shown in Table 4a,b. In these models, the dependent variables used for the regression equations were indicators of muscle fatigue, cell membrane disruption, and loss of muscle mass. The predictive variables were testosterone and the testosterone/estrogen ratio. We can consider that in our regression equations within a multicausal model, testosterone was the main protective factor against the muscle damage and fatigue caused by severe exhaustion after the ultramarathon. This observed phenomenon, as can be seen in Table 3 was more evident in women who were not receiving hormonal contraception.

## 4. Discussion

The results from the present study highlight testosterone levels as a predicting factor of loss of strength and muscle damage after ultra-endurance events in female athletes. Moreover, the T/E ratio suggests a possible indirect role of estrogens in reducing exercise-induced muscle damage and improving the recovery of athletes. 

Menstrual cycle disturbances are common in women performing prolonged strenuous exercise at competitive levels. These disturbances have traditionally been considered to be secondary to energy deficiency and a suppressed hypothalamic pituitary–gonadal axis [32,33]. Amenorrhea, estrogen deficiency, and low energy availability are associated with a rapid loss of bone mass and an elevated risk of musculoskeletal injuries [34]. Moreover, under low energy availability, LH pulsatility is abolished and levels of testosterone are low [15]. However, not all female athletes who exhibit disturbances in their menstrual cycle present with this catabolic situation. In this respect, Hagmar [13] recently demonstrated that essential hyperandrogenism, such as that associated with PCOS, represents an alternative cause of menstrual disorders in athletes. Interestingly, the levels of testosterone in women diagnosed with PCOS are within the upper physiological limits. Furthermore, these athletes present an increased anabolic state, as they have more muscle mass and better bone mineral density as compared to other female athletes [35]. 

Testosterone is one of the most potent naturally secreted androgenic anabolic hormones. In muscle, it stimulates protein synthesis with an anabolic effect [36] and inhibits protein degradation with an anti-catabolic effect [37]. This leads to increased protein synthesis and decreased protein breakdown [38]. Traditionally, this compound has been abused by elite athletes to achieve these anabolic effects. In women, although testosterone is not generally considered to be a primary anabolic hormone, it nonetheless has a powerful effect on muscle tissue [38]. In fact, recently, Hirschberg et al. [14], in a double blind, randomized, placebo-controlled study, investigated the effects of moderately increased testosterone concentrations on physical performance in young women, finding that the supplemented group showed increased performance and muscle strength.

Our study showed that the participants’ basal testosterone levels were within the low range of normal testosterone concentrations. This finding is in accordance with previous published data regarding gender-specific differences in sex hormones as a result of ultra-endurance exercise [39,40], and is better explained by the hypometabolic state hypothesis since this endocrine disturbance is characterized by low testosterone levels [4]. Moreover, in our results, participants with higher testosterone levels showed less strength loss and muscle membrane disruption after ultra-endurance exercise. This finding was supported by the strong negative correlations found between levels of this hormone; indirect markers of muscle damage, such as CK (at the finish line and 24/48 h later) and LDH (24/48 h later); and the evolution of the HG test before and after the ultra-trail race. These results were especially relevant for testosterone levels in the group not receiving exogenous hormone supplementation through HC. In this group, the variability explained by our models increased dramatically, indicating that levels of testosterone might be able to predict variability of 68.9%, 70.2%, 55.6%, and 65.8% for CK, LDH, HG, and the MM delta value, respectively. In accordance with the criterion proposed by Cohen [31], our regression analysis within a multicausal model demonstrates that testosterone is the main protective factor against muscle damage and fatigue after severe exertion on running an ultramarathon.

In contrast, no effect of basal estrogen levels was observed. However, the T/E ratio positively correlated with the loss of muscle strength and the increase in muscle membrane disruption immediately after the race and 48 h later. These results suggest an indirect protective role of estrogens in female ultramarathon runners and mark the relevance of sex hormone interactions. In accordance with our proposed role of estrogens, Hansen [41] described a similar estrogen-elicited mechanism that increases sensitivity to training, reducing exercise-induced muscle damage and improving recovery. Regarding the T/E ratio, some other authors have considered this relationship to be an over-training predictor associated with fatigue in male athletes [17,18]. To date, there is no previous literature regarding this ratio in female athletes, however, in our study, the participants showed a much lower ratio than male athletes. This was an expected result since estrogen is the primary female sex hormone. Further investigation is necessary to confirm this novel result in female runners and its physiological implications in ultra-endurance sports performance.

Regarding the use of HC methods, in our study, we did not find differences between women who used contraceptives and those who did not in relation to strength and muscle damage after ultra-endurance exercise, indicating that no added benefit is provided by adjusting the menstrual cycle with HC. As expected, estrogen levels were significantly decreased in this group because of HPA downregulation. However, further studies are necessary to confirm the effect of different types of HC upon HPA axis inhibition in ultra-trail runners. We did not find differences in baseline hematological variables and serum iron levels in our study groups. In line with this, it has been reported that resistance and exercise performance are not affected by changes in the menstrual cycle resulting from the use of contraceptives [42,43,44,45]. However, other authors have highlighted the possible benefit of HC methods for reducing menstrual blood loss and increasing serum iron levels, which may contribute to a greater oxygen-carrying capacity [46]. 

## 5. Limitations

This study yielded important results on the role of testosterone in muscle physiology in female athletes. However, it has some limitations. The first is in regards to the HC type, dose, and route of administration. Although functionally these contraceptives target anovulation, their effects on the HPA axis differ. Secondly, it is extremely difficult to obtain large samples of women participating in 115-kilometer races. In this context, having achieved a sample of *n* = 19 female athletes is notable. However, it would be desirable to expand the number of similar studies to obtain larger and more representative samples and contrast the results. Finally, there is the impossibility of obtaining volunteer participants at the same point in their menstrual cycle. These difficulties condition the methodological design of the study. 

## 6. Conclusions

Even though the current literature suggests that fluctuations in female steroid hormones throughout the menstrual cycle do not affect muscle strength, in our research, we found a relationship between these variables and endogenous serological basal testosterone serum levels. 

The T/E ratio may be a good predictor of muscle damage and post-race recovery, but this needs to be studied in greater detail in future investigations.

We did not find differences between HC users and non-HC users in terms of hematological variables, strength, systemic inflammation, or muscle damage after running an ultra-trail race.

A practical implication of these findings would be that in female athletes participating in specific endurance sports, such as ultra-trail racing, it is not necessary to adjust the menstrual cycle using HC to maximize competitive capacity, as this provides no added benefit.

However, depending on the menstrual cycle phase, there are physiological fluctuations in endogenous testosterone levels that condition greater fatigability and muscle damage after training. This factor could be taken into consideration in the design of training guidelines. Moreover, the present findings could provide a basis for a new approach to improve recovery patterns after ultra-long-distance events depending on the menstrual cycle phase.

## Figures and Tables

**Table 1:** Descriptive data on sex hormones and variables of muscle fatigue, loss of muscle mass, and injury to muscle tissues (mean ± SD).

	Baseline Data	Finish Line	24 h Post-Race	48 h Post-Race
Estradiol (pg/mL)	89.06 ± 87.99			
Testosterone (ng/dL)	19.34 ± 9.34			
T/E ratio	1.25 ± 1.99			
CK (ui/L)	137.59 ± 54.73	5075.76 ± 3871.18 *	2036.61 ± 1389.83 * ^#^	905.30 ± 534.95 * ^#^
LDH (ui/L)	185.88 ± 25.29	380.59 ± 112.80 *	320.93 ± 93.97 * ^#^	303.38 ± 86.67 * ^#^
MM (%)	39.20 ± 3.45	38.00 ± 4.86 *		
HG (kg)	31.33 ± 3.69	28.67 ± 4.10 *		
SJ (cm)	20.76 ± 2.72	18.47 ± 2.38 *		

Abbreviations: T/E ratio: testosterone/estradiol ratio; CK: creatine kinase; LDH: lactate dehydrogenase; MM: muscle mass; HG: hand grip; SJ: squat jump. * Significantly different from the preceding time point (*p* < 0.05); # significantly different from the pre-race value (*p* < 0.05).

**Table 2:** Spearman correlations between sex hormones, muscle fatigue variables, and indirect markers of muscle damage.

	Estradiol Correlation *r* Value/*p* Value	Testosterone Correlation *r* Value/*p* Value	T/E Ratio Correlation *r* Value/*p* Value
Δ CK finish line	−0.199/0.514	−0.674 */0.012	0.435/0.137
Δ CK 24 h	−0.324/0.280	−0.652 */0.016	0.319/0.288
Δ CK 48 h	−0.428/0.145	−0.619 */0.024	0.134/0.662
Δ LDH finish line	−0.065/0.832	−0.541/0.056	0.581 */0.037
Δ LDH 24 h	−0.114/0.712	−0.585 */0.036	0.595 */0.032
Δ LDH 48 h	−0.147/0.631	−0.615 */0.025	0.620 */0.024
Δ MM finish line	0.202/0.508	−0.466/0.109	0.625 */0.02
Δ SJ finish line	−0.206/0.520	0.132/0.683	−0.065/0.841
Δ HG finish line	−0.472/0.104	−0.755 */0.003	−0.034/0.912

Abbreviations: Δ CK: fold increase creatine kinase; Δ LDH: fold increase lactate dehydrogenase; Δ MM: fold increase muscle mass; Δ SJ: fold increase squat jump; Δ HG: fold increase hand grip. * Significantly different (*p* < 0.05).

**Table 3:** Relation between contraceptive groups and study variables.

	No Hormonal Contraceptive Methods	Hormonal Contraceptive Methods	*p*-Value/d Cohen
**Baseline ferritin (ng/mL)**	24.11 ± 11.37	24.72 ± 8.75	0.912
**Red blood cells (ng/mL)**	4.649 ± 0.25	4.90 ± 0.29	0.084
**Hemoglobin (g/dL)**	14.24 ± 1.31	14.41 ± 1.23	0.792
**Baseline red blood cells (%)**	43.05 ± 3.14	43.78 ± 2.91	0.645
**Estradiol (pg/mL)**	129.20 ± 93.85	33.10 ± 32.83	0.012 */2.16
**Testosterone (ng/dL)**	21.58 ± 10.37	16.79 ± 7.57	0.313
**T/E ratio**	0.86 ± 1.78	1.97 ± 2.33	0.290
**CK (IU/L) baseline**	147.80 ± 66.65	126.83 ± 31.012	0.485
**CK (IU/L) finish line**	5640.55 ± 4572.41	3072.91 ± 940.02	0.303
**CK (IU/L) 24 h**	2202.62 ± 1613.55	1392.00 ± 415.22	0.356
**CK (IU/L) 48 h**	921.25 ± 563.60	646.50 ± 197.30	0.246
**LDH (IU/L) baseline**	187.50 ± 31.28	181.00 ± 14.71	0.643
**LDH (IU/L) finish line**	393.36 ± 132.56	327 ± 41.45	0.366
**LDH (IU/L) 24 h**	331.37 ± 117.06	290.75 ± 37.50	0.522
**LDH (IU/L) 48 h**	309.62 ± 110.85	286.50 ± 28.06	0.696
**MM (%) baseline**	43.85 ± 3.65	45.26 ± 3.16	0.439
**MM (%) finish line**	43.36 ± 5.12	44.27 ± 4.86	0.439
**Δ SJ finish line**	−0.16 ± 0.07	−0.06 ± 0.14	0.112
**Δ HG finish line**	−0.10 ± 0,12	−0.01 ± /0.10	0.771

Abbreviations: CK: creatine kinase; LDH: lactate dehydrogenase; MM: muscle mass; Δ SJ: fold increase squat jump; Δ HG: fold increase hand grip. * Significantly different (*p* < 0.05).

**Table 4:** Multivariate analysis.

**a. Linear regression models.**				
**Model**	**R^2^ Adjusted**	**Standardized Coefficients Beta**	**Standard Error**	**F (*p*)**
Dependent variable: Δ CK finish line Covariables: Testosterone.	0.405	–0.674	2.69663	9.151 (0.012)
Dependent variable: Δ LDH finish line Covariables: T/E ratio.	0.479	–0.579	0.36986	6.507 (0.015)
Dependent variable: Δ HG finish line Covariables: Testosterone	0.531	–0.755	0.19891	14.567 (0.003)
Dependent variable: Δ MM finish line Covariables: T/E ratio.	0.335	0.625	3.96370	7.056 (0.022)
**b. Linear regression models for women not receiving hormonal contraception (*n* = 11; 66.7%).**				
**Model**	**R^2^ Adjusted**	**Standardized Coefficients Beta**	**Standard Error**	**F (*p*)**
Dependent Variable: Δ CK finish line Covariables: Testosterone.	0.689	–3.222	0.744	18.756 (0.003)
Dependent Variable: Δ LDH finish line Covariables: Testosterone.	0.702	–0.046	0.31096	19.819 (0.003)
Dependent Variable: Δ HG finish line Covariables: Testosterone	0.556	–0.022	0.19891	8.777 (0.021)
Dependent Variable: Δ MM finish line Covariables: T/E Ratio.	0.658	0.370	2.9994	16.371 (0.005)

Abbreviations: Δ CK: fold increase creatine kinase; Δ LDH: fold increase lactate dehydrogenase; T/E ratio: testosterone/estrogen ratio; Δ HG: fold increase hand grip; Δ MM: fold increase muscle mass).

## Data Availability

All data generated or analyzed during this study are included in this published article.
